# Managing Potential Australian Bat Lyssavirus (ABLV) Exposure in Regional Queensland: A Mixed-Methods Investigation of Epidemiology and Operational Challenges

**DOI:** 10.3390/epidemiologia7050143

**Published:** 2026-10-13

**Authors:** Ho Yeung Lam, Luke Wallington, Ben Brimblecombe, Shalomie Shadrach, Teresa McGorm

**Affiliations:** Darling Downs Public Health Unit, Darling Downs Hospital and Health Service, Queensland Health, Toowoomba, QLD 4350, Australia

**Keywords:** Australian bat lyssavirus, post-exposure prophylaxis, infectious disease epidemiology, mixed-methods study, regional Queensland, rabies virus

## Abstract

**Background/Objectives**: Australian bat lyssavirus (ABLV) is a rare but fatal zoonotic infection transmitted from infected bats. In Queensland, Australia, all potential human exposures should be reported to local public health units for assessment and post-exposure prophylaxis (PEP). Following a marked increase in notifications in 2025, the Darling Downs Public Health Unit (DDPHU), serving the Darling Downs and South West regions of Queensland, evaluated the epidemiology and operational challenges using a retrospective mixed-methods design. **Methods:** Data of all potential ABLV exposure notifications within the DDPHU catchment between 2011 and 2025 were collected from existing databases to describe demographic, temporal, spatial, and case management outcomes. Staff meeting records were analysed using framework analysis to identify operational challenges. **Results:** There were 237 potential exposure notifications, with annual counts fluctuating and peaks noted in 2013 and 2025. Notifications showed a seasonal pattern, peaking in spring and summer. Demographic characteristics of cases remained similar over time. Exposures were concentrated in the eastern part of the catchment, with no significant spatio-temporal clustering. Intentional contact with bats remained the leading cause of exposure. Over the study period, the proportion with documented PEP completion increased, as did timeliness of notification and PEP initiation. However, staff management time per case increased. Key operational challenges included the time-critical nature of case management, atypical exposure scenarios, and logistical complexity in arranging PEP across multiple providers. Availability of pre-positioned PEP at selected hospitals was perceived by staff to reduce workload. **Conclusions:** Although potential exposures aligned with known bat distribution and seasonal activity, the 2025 surge resulted in greater workload. Measures, including improved public health messaging, expanded pre-positioned PEP, and local protocols for atypical scenarios, may optimise case management and support workforce sustainability. These findings also highlighted the importance of accessible PEP and effective service coordination for timely management of potential lyssavirus exposures in non-urban settings.

## 1. Introduction

Australian bat lyssavirus (ABLV) is a zoonotic virus closely related to the rabies virus [1]. First described in 1996, ABLV is unique to Australia and has been detected in a variety of bat species, including flying foxes (fruit bats) and insectivorous microbats [2]. All Australian bat species have the potential to carry and transmit ABLV [3].

ABLV can be transmitted to a person through bites, scratches, or mucosal exposure to saliva or neural tissue from an infected bat. Although rare, ABLV infection in humans is extremely serious, with all four reported cases in Australia to date being fatal [4,5,6,7].

Although the estimated prevalence of ABLV in wild bat populations is low at approximately 0.5% [8], it may be higher among sick or behaviourally abnormal bats [9], which are also more likely to come into contact with humans. Given this uncertainty, all bats in Australia are regarded as potentially infectious, and thus any bite, scratch, or mucosal exposure from a bat should be assessed to determine whether it represents a potential ABLV exposure and if post-exposure prophylaxis (PEP) is indicated [10]. PEP for ABLV consists of a course of rabies vaccination, with or without the local and systemic administration of human rabies immunoglobulin (HRIG), based on in vitro evidence demonstrating their cross-neutralisation and effectiveness against ABLV [11,12,13,14].

**Figure 1:**
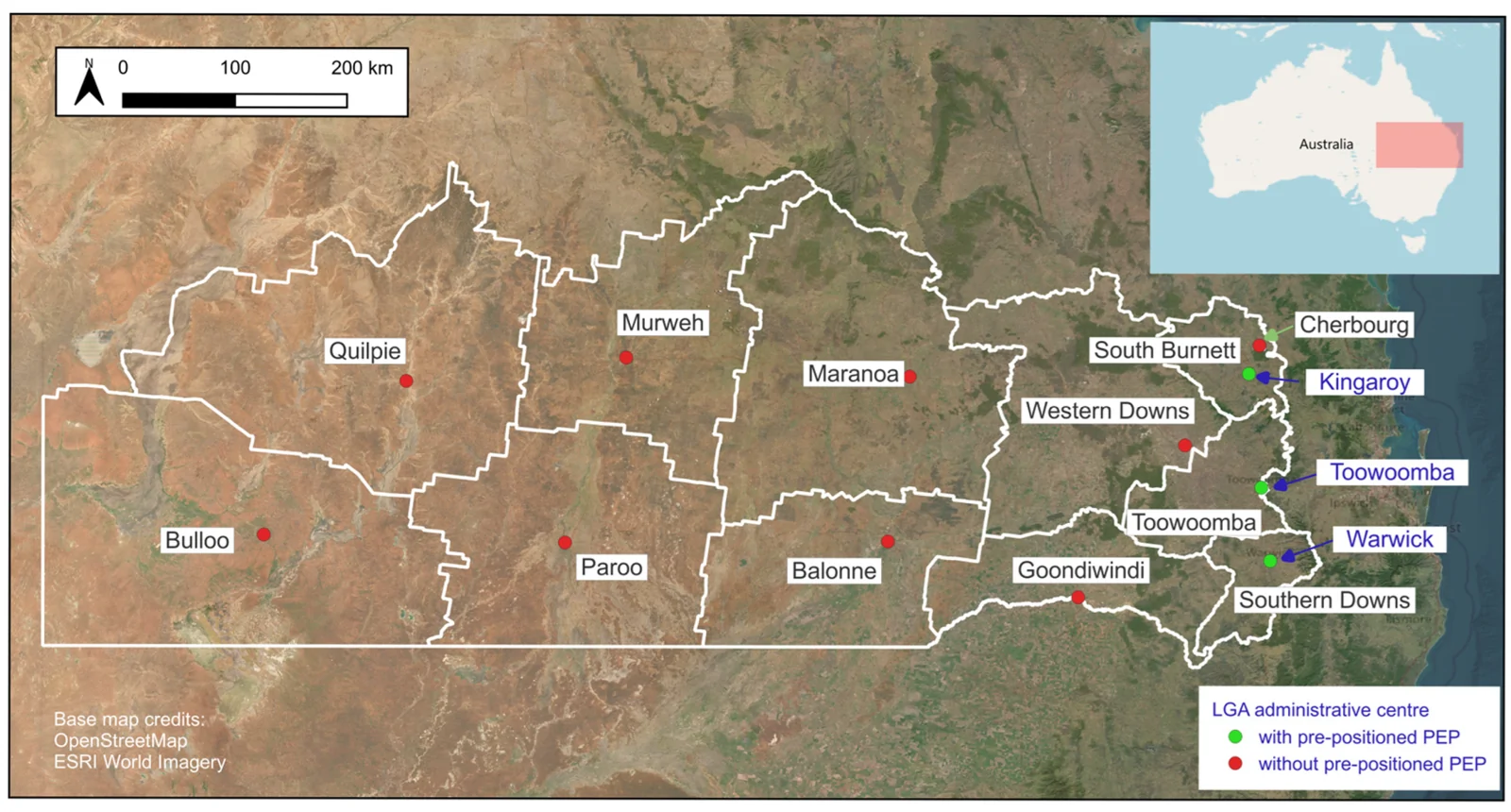
Local government areas under study (black font), with locations where pre-positioned post-exposure prophylaxis (PEP) is available (blue font).

In Queensland, Australia, flying foxes are concentrated along coastal areas and nearby inland regions, whereas insectivorous microbats have a much broader distribution, with some species occurring across the entire state [15]. Potential ABLV exposure has been a notifiable condition under the Public Health Act of Queensland since 2005. Clinicians are required to notify the local public health unit (PHU) when a potential exposure is identified. In the Darling Downs and South West regions of Queensland, notifications are managed by the Darling Downs Public Health Unit (DDPHU), which serves a population of over 320,000 across a large geographic area encompassing regional, rural and remote communities from 13 local government areas (LGAs) [16] (Figure 1). This region is characterised by a transition from subtropical climate in the east through grassland climate to desert climate in the west, with hot, dry summers associated with extreme heat events and cool winters [17].

Management of potential ABLV exposure cases by PHUs is guided by the Communicable Diseases Network Australia (CDNA) National Guidelines [10]. Upon notification, PHU staff undertake a risk assessment based on the patient’s clinical status and the circumstances of exposure to determine whether the event meets the case definition for a potential ABLV exposure and whether PEP is indicated. Where required, arrangements are made for the patient to receive PEP as soon as possible at the nearest public hospital emergency department (ED) where pre-positioned PEP is available (in Toowoomba, Warwick, and Kingaroy). In some circumstances, such as when patients reside in remote areas far from facilities with pre-positioned PEP, the initial doses may be transported to the patient’s nearest health facility for administration. Subsequent doses of rabies vaccine will be administered through the patient’s general practitioner (GP) or public hospital. PHU staff then follow up with the patient and vaccine providers to ensure PEP has been completed according to schedule and that vaccination details are accurately recorded in the Australian Immunisation Register (AIR).

Beyond managing notifications, the role of DDPHU includes health promotion and risk communication aiming to reduce the risk of ABLV exposure in the community. This includes public messaging through media and educational initiatives that emphasise avoiding contact with bats and seeking medical advice following bat exposure. DDPHU also works closely with primary healthcare providers and hospitals to support timely notification, coordination, and effective case management.

In 2025, there was a marked increase in notifications of potential ABLV exposure in Queensland. For DDPHU, more than thirty cases were recorded in 2025, representing more than a threefold increase compared with 2024 and the highest annual number reported in the preceding ten years. In contrast, notifications across Queensland increased by 13% over the same period. This disproportionate rise in notifications to DDPHU has resulted in increased workload demand for its staff, involving time-intensive activities such as risk assessment, coordination with clinicians, provision of health advice, and the arrangement and follow-up of PEP. A review of these notifications, together with an examination of current operational challenges faced by PHU staff, will provide valuable insights into both potential changes in exposure patterns and the practical implications of managing these notifications. Such findings can also inform service planning and health promotion strategies. Findings from this study may also inform broader public health responses to potential lyssavirus exposures in other non-urban settings.

## 2. Materials and Methods

### 2.1. Study Design and Objectives

This is a retrospective mixed-methods study combining quantitative analysis of potential ABLV exposure notifications from 2011 to 2025 and qualitative analysis of DDPHU meeting records to examine operational challenges in potential ABLV exposure case management. This study was approved by the Darling Downs Health Human Research Ethics Committee (LNR/2026/QTDD/125628). The objectives of the study are summarised in Table 1.

### 2.2. Data Collection and Analysis

#### 2.2.1. Quantitative Data

Records of potential ABLV exposure cases notified to DDPHU were extracted from the Notifiable Conditions System (NoCS), a state-wide database used by PHUs in Queensland to record demographic, exposure, and key management information for notifiable conditions. These data were supplemented with additional case management details extracted from internal DDPHU records.

Eligible cases included all confirmed potential ABLV exposure notifications reported to DDPHU between 1 January 2011 and 31 December 2025, where the exposure occurred within the DDPHU catchment area. A confirmed potential exposure was defined in accordance with CDNA guidelines as any notification involving a bite or scratch from a bat, mucous membrane or broken skin contact with bat saliva or neural tissue, or situations involving contact with bats where bites or scratches may not have been apparent [10].

For each eligible case, data were extracted on demographics (age, sex, and First Nations status), exposure characteristics (location, date and time, bat type, nature and circumstances of exposure), PEP management (whether and what PEP was indicated, completion status, and date receiving first rabies vaccine dose and HRIG, where applicable), and the documented time spent by DDPHU staff managing each case. As part of the standard workflow, staff recorded the minutes spent on each individual case-management task in the DDPHU system. Total management time for each case was calculated by summing the time recorded for all tasks under that case. PEP completion was defined as documented completion of the recommended PEP course among cases for whom PEP was required.

Year-specific population denominator data at LGA levels were retrieved from the Australian Bureau of Statistics. Notifications were presented as annual counts and rates per 100,000 population, with annual rates calculated as the number of potential ABLV exposure notifications in each LGA in each calendar year divided by the corresponding population. Data describing the geographic distribution of different Australian bat species were obtained from the BatMap database [15].

Data analyses were conducted using R (version 4.5.1) [18]. Data manipulation and visualisation were performed using the tidyverse [19], sf [20], and tmap [21] packages. Descriptive statistics were used to summarise the epidemiology of notifications, including counts, proportions, and distributions of key demographic and exposure characteristics across three 5-year intervals (2011–2015, 2016–2020, 2021–2025). Missing data for categorical variables were classified as “Unknown” and retained in inferential statistical tests. Observations with missing values for continuous variables were excluded from the relevant statistical analyses. Temporal trends were examined at annual and monthly levels. To assess seasonality and differences in seasonal patterns between periods, monthly notification counts were aggregated across the three 5-year study periods and analysed using Poisson regression models including study period, month, and their interactions. Model comparisons were performed using likelihood-ratio tests, and overdispersion was assessed using the Pearson dispersion statistic. Spatial patterns of exposure were explored using geospatial visualisation, supplemented by bat species distribution data from Batmap. Where appropriate, inferential statistical tests (e.g., chi-square tests) were applied to assess differences in the distribution of cases across categories between the three study periods. Spatio-temporal clustering was assessed using SaTScan (version 10.3.3), a free and open-source software package designed to detect disease clusters through scan statistics [22]. The rsatscan package was used to interface with SaTScan in R [23]. A retrospective space–time permutation scan statistic with monthly temporal aggregation was used to identify high-rate clusters (≥5 cases) within circular windows of up to 50 kilometres. The maximum spatial cluster sizes were set to 10% of the population at risk, consistent with a previous study of potential ABLV exposures in Queensland [24], and the maximum temporal cluster size was set to six months. These parameter choices were guided by the dispersed geography of the study region, seasonality of exposure events, and the need to balance sensitivity for cluster detection and statistical stability given the relatively small number of cases. Statistical significance was assessed using 999 Monte Carlo replications (*p* < 0.05).

#### 2.2.2. Qualitative Data

Data on operational challenges encountered by DDPHU staff were extracted from records of Continuous Quality Improvement (CQI) meetings, attended by medical and nursing staff to review operational issues and optimise management of notifiable conditions. All CQI meeting records were reviewed, and items relevant to potential ABLV exposure management were extracted for analysis.

Qualitative analysis was conducted using NVivo (version 15.3.2, Lumivero). A document analysis approach [25,26] was undertaken using a framework analysis methodology [27]. Analysis followed the five stages of familiarisation, development of the analytical framework, indexing, charting, and mapping and interpretation. The analytical framework was informed by the Systems Engineering Initiative for Patient Safety (SEIPS) model [28] and comprised four domains: person, processes and tasks, technology and tools, and organisation. Relevant data were identified within the meeting records and then coded deductively under these domains by the lead author. Codes within each domain were then iteratively reviewed, compared, and synthesised as themes to identify key operational challenges. To enhance the credibility of the findings, preliminary themes developed were presented to DDPHU staff for review and validation, with their feedback incorporated in the final themes where appropriate. Qualitative findings were subsequently integrated with quantitative results to support interpretation of observed trends and inform implications for service planning and public health practice.

## 3. Results

### 3.1. Quantitative Findings

During the study period, 259 potential ABLV exposure notifications were reported to DDPHU. Of these, 256 were classified as confirmed potential exposures. A total of 237 cases occurred within the DDPHU catchment area and thus were included in the analysis.

**Figure 2:**
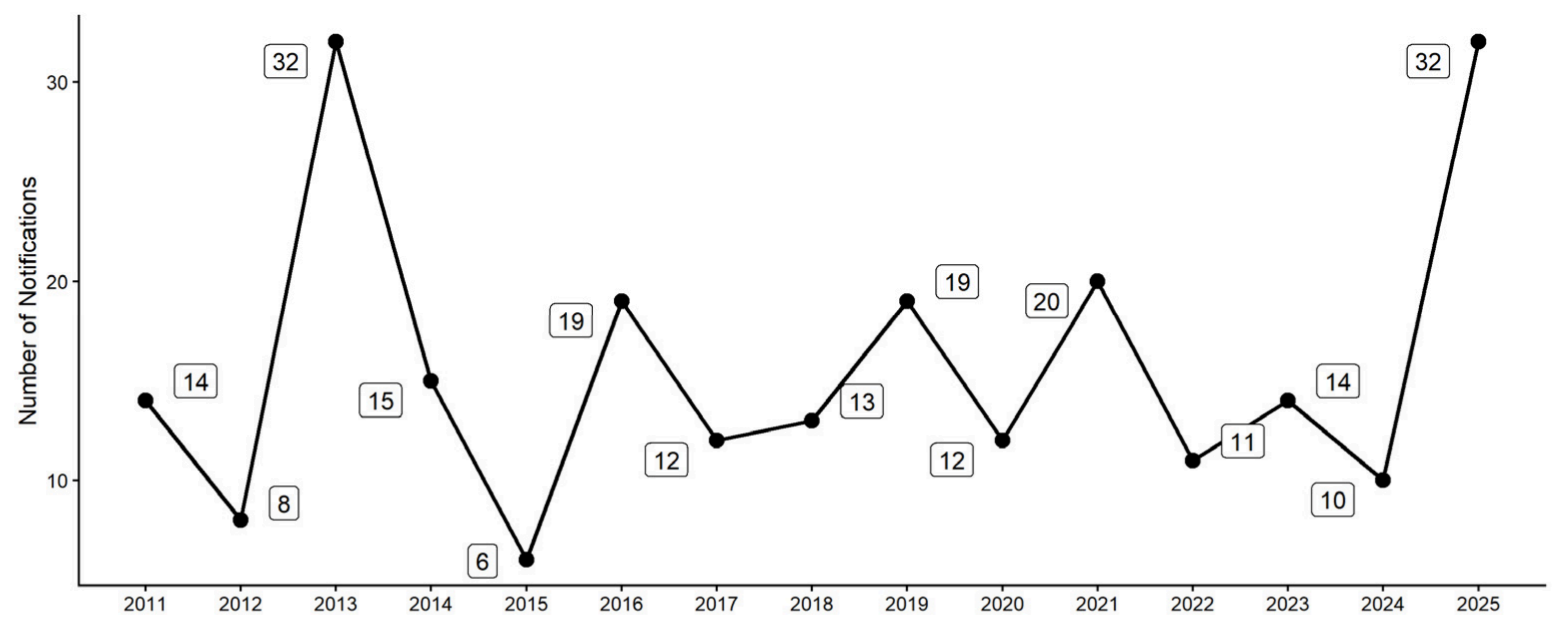
Annual number of potential ABLV exposure notifications, 2011–2025.

The annual notification counts varied considerably, ranging from 6 to 32 cases (Figure 2). Between 2011 and 2015, notifications were generally low (6 to 15 per year), except for a surge in 2013 (32 cases). It then fluctuated between 10 and 20 cases per year between 2016 and 2024, before increasing again in 2025 (32 cases).

**Figure 3:**
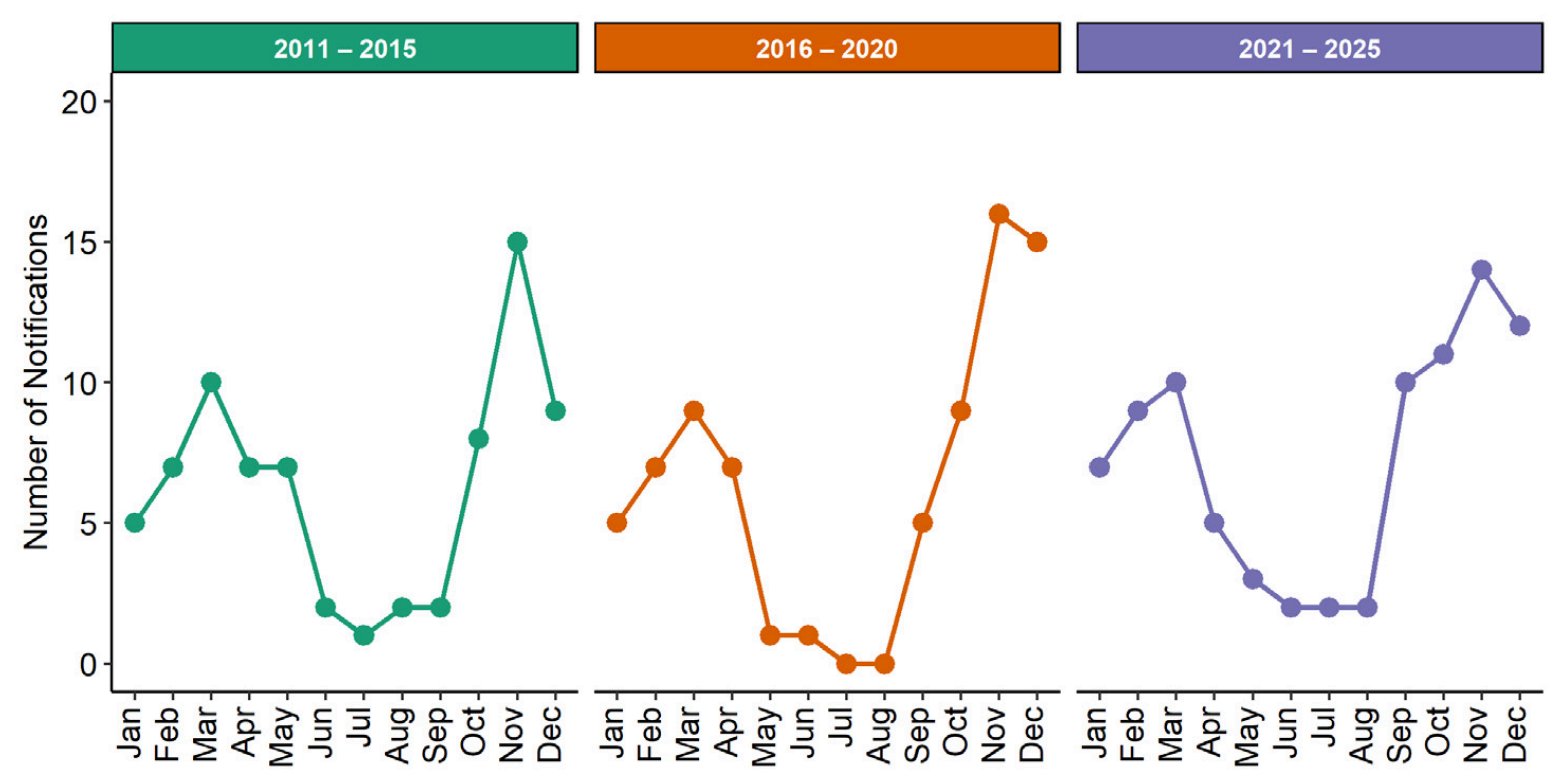
Aggregated monthly notifications of potential ABLV exposures, 2011–2025, across three study periods.

Notification by month demonstrated a clear seasonal pattern (Poisson regression likelihood ratio test, *p* < 0.001). The model including study period and month showed no evidence of overdispersion (Pearson dispersion statistic = 0.71). There was no evidence that the seasonal pattern differed between the three study periods (period–month interaction, *p* = 0.81). Counts were generally higher during spring and summer, peaking in November and December and remaining elevated through March, and were lowest during winter months, particularly from June to August (Figure 3).

Across the study period, the median age of exposed individuals was 46 years (IQR 29 to 59), with an equal sex distribution (50% male, 50% female). More than 90% of cases involved non–First Nations individuals, while 3.0% identified as First Nations. No statistically significant temporal changes in these demographic characteristics were identified (Table 2).

Most exposures occurred during the morning, afternoon, and evening in similar proportions, while overnight exposures remained uncommon throughout the study period. More than half of all exposures resulted from intentional contact with bats by members of the general public, while the proportion of accidental contact declined over time, from 31% in 2011–2015 to 14% in 2021–2025. In contrast, occupational exposures (e.g., bat carers rescuing bats) increased from fewer than 10% to 28% (Table 2).

Flying foxes were the most frequently implicated bat type throughout the study period, while exposure involving microbats declined. The majority of exposures remained bites or scratches from bats, although there was an increasing proportion of cases in recent years that involved either no observable wounds or pre-existing skin wounds after direct bat contact (Table 2).

**Figure 4:**
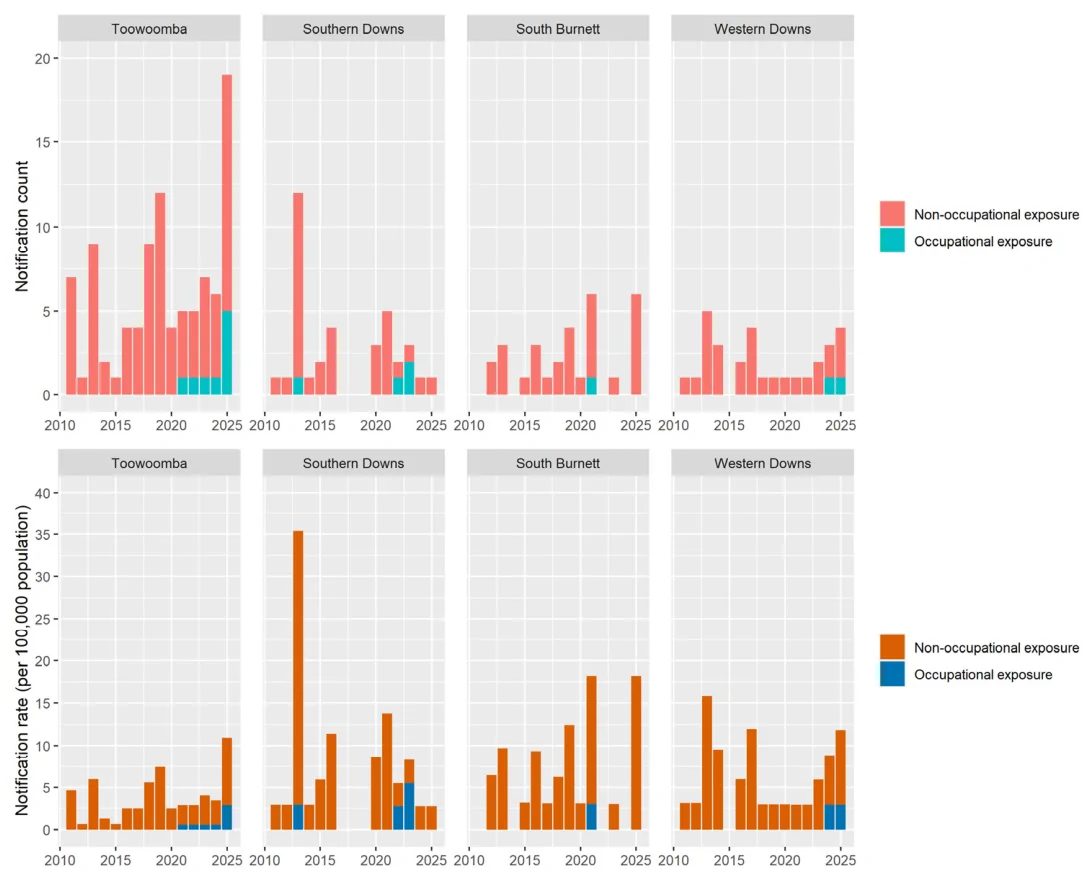
Notification counts and potential exposure notification rates across four LGAs, 2011–2025.

Geographically, more than two-thirds of exposures occurred in the four easternmost LGAs of DDPHU catchment, namely Toowoomba, Southern Downs, South Burnett, and Western Downs. Toowoomba contributed the largest number of cases and showed an increase in both the number and proportion of notifications over time, from 21 (28%) in 2011–2015 to 34 (45%) in 2016–2020 and 42 (48%) in 2021–2025 (Table 2). While Toowoomba recorded the highest number of notifications, South Burnett had some of the highest notification rates (ranging from 11.4 to 35.4 per 100,000 population) among the four LGAs throughout the study period. In 2025, Southern Downs experienced reductions in both notification count and rate (one notification corresponding to 2.8 per 100,000 population), whereas Toowoomba, South Burnett, and Western Downs showed increases in both compared to previous years (Figure 4).

**Figure 5:**
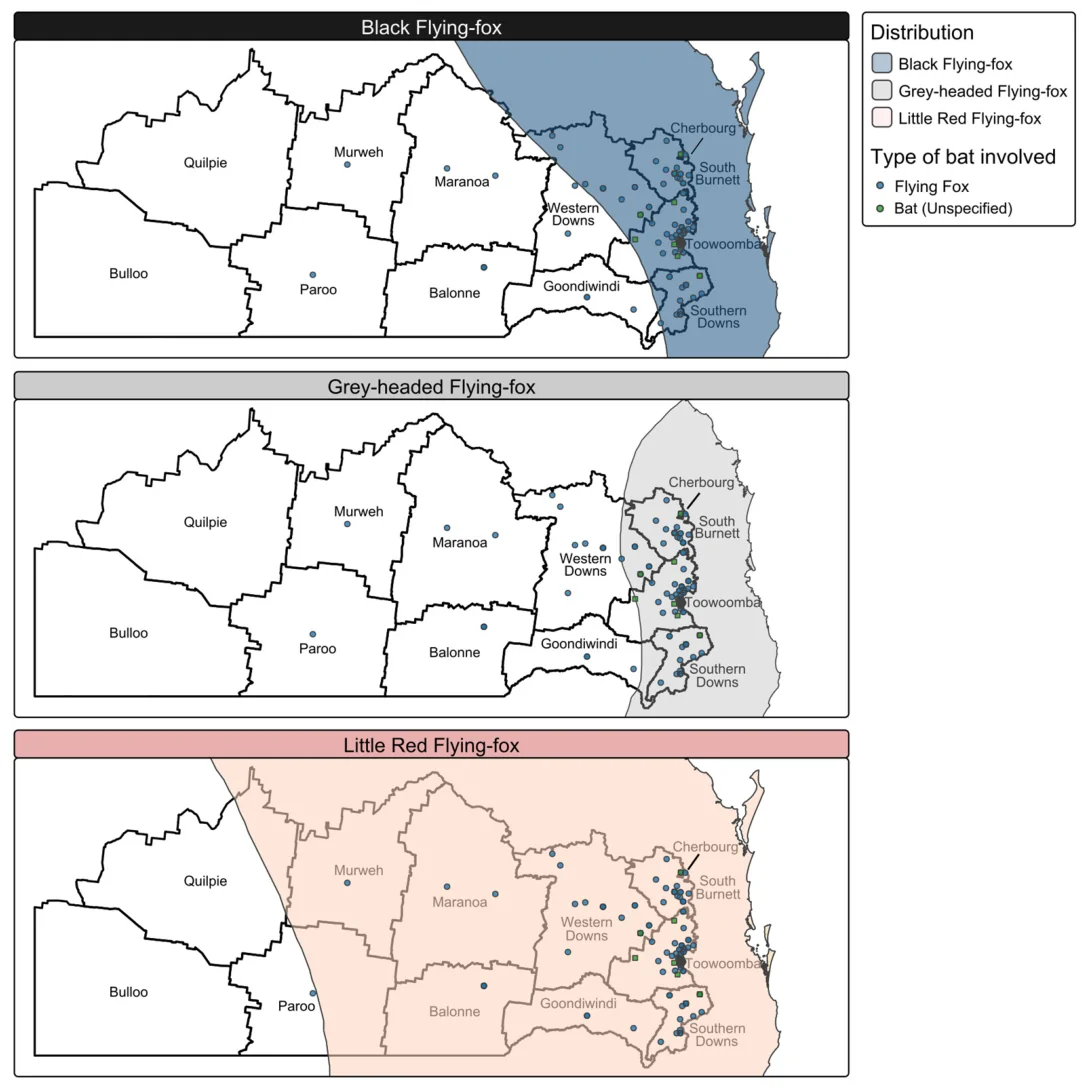
Geographical distribution of potential ABLV exposures involving flying foxes or bats (unspecified), overlaid with flying fox distribution. Note: Microbat distributions are not shown due to their widespread distribution across the study area; dots indicate suburb centroids, not exact exposure sites.

Geospatial mapping indicated that the locations of exposure involving flying foxes or bats (unspecified) closely overlapped with areas known to be inhabited by flying foxes (Figure 5). Spatio-temporal analysis using retrospective space–time permutation scan statistics did not identify any statistically significant clusters.

Testing of bats involved in the exposure incidents was uncommon (less than 10%) and declined over time. An increasing proportion of notifications were assessed as requiring PEP, with all cases notified during 2021–2025 deemed to require PEP. Between 2011 and 2015, cases requiring rabies vaccinations required either two, four or five doses. From 2016 onwards, nearly all cases required either two or four doses of rabies vaccine, and the proportion of cases reported to have received at least one rabies vaccine dose increased substantially over time. Although the proportion of cases requiring HRIG decreased during 2021–2025 compared with previous periods, among those for whom HRIG was indicated, the proportion reported as having received HRIG increased from 2016 onwards. The proportion of documented completion of recommended PEP increased from 29% in 2011–2015 to 89% in 2016–2020 and 95% in 2021–2025, noting that the proportion of unknown PEP outcomes also decreased substantially over the same periods, from 57% to 5.3% and 1.1%, respectively. Two cases refused PEP during 2021–2025. One cited a perceived lack of ABLV infection risk, and the other believed that vaccination was unnecessary. The proportion of cases not requiring PEP because the bat involved was tested negative decreased from 12% in 2011–2015 to 0% in 2021–2025. Notably, more than half of the cases’ PEP outcome was not recorded during 2011–2015 (Table 2).

Timeliness of case management improved over time. The median interval from exposure to notification decreased from five days in 2011–2015 to one day in later periods, while the median time from exposure to first rabies vaccine administration decreased from two days to one day. The median time from exposure to HRIG administration remained one day with no temporal change. The median time spent by DDPHU staff managing each notification decreased from 110 min (20/75 observations) in 2011–2015 to 85 min (73/75 observations) in 2016–2020, before increasing significantly to 160 min (87/87 observations) in 2021–2025 (Table 2).

### 3.2. Qualitative Findings

Meeting minutes from three CQI meetings conducted in January 2026 were included in the analysis. Across the three meetings, three public health doctors and seven public health nurses contributed to the discussions, drawing on their case management experiences from the preceding 2–3 years. A total of seven themes were identified across the four SEIPS domains (Table 3).

PHU staff consistently described the management of potential ABLV exposure notifications as a time critical process that often required reprioritising other duties to ensure rapid assessment and timely access to PEP for the patients. The circumstances of exposure were highly variable and thus required case-by-case evaluation rather than routine application of standard protocols. Although existing guidelines covered many common scenarios, there were situations that were not explicitly addressed, such as cryptogenic exposures, exposures involving bilateral wounds, no visible wound, or contact over pre-existing wounds, and the use of rabies serology to inform deviations from recommended vaccine schedules, all of which required careful deliberation and professional judgement.

Case management routinely involved extensive communication with patients to clarify the circumstances of exposure and, when a potential exposure was deemed plausible, coordination with healthcare providers to facilitate timely initiation of PEP. Staff described these processes as time-demanding, particularly when clarifying exposure details and arranging PEP with hospital EDs and general practices. The availability of pre-positioned rabies vaccines and HRIG at selected EDs was universally regarded as highly facilitative and convenient. However, arranging PEP delivery to other health facilities remained necessary, particularly for patients living in outer regional or rural areas who often faced barriers travelling to EDs with pre-positioned rabies vaccines and HRIG. Although current guidelines mentioned bat testing as a potential tool to inform PEP decisions, its logistical and financial requirements had limited its routine use.

Once PEP was initiated, ensuring completion of the vaccination schedule required regular follow-up. Challenges arose when patients were in other locations (e.g., for work or travel) during the course of PEP, which was relatively common given that the regimen often spans at least two weeks. This necessitated engagement with multiple providers and coordination of vaccine delivery to different sites. Adhering to the strict dosing schedules of rabies vaccines could also be difficult at times when scheduled doses fell on weekends when availability of their GPs was limited.

Documentation and record management represented another key component of case management. Vaccinations administered in public hospitals required manual entry into AIR, in contrast to GP settings where reporting to AIR is commonly automated through practice management software. PHU staff frequently needed to follow up with hospitals to confirm vaccine administration details and AIR upload, which added to the administrative workload.

## 4. Discussion

### 4.1. Epidemiology of Potential ABLV Exposures

Throughout the study period, annual notifications of potential ABLV exposure varied considerably, with a notable increase in 2013 and 2025. Nevertheless, there were no significant changes in demographic characteristics including age, sex, or First Nations status. Of note, First Nations people accounted for 3.0% of notifications, while representing 7.1% of the population in the study area [29,30]. In addition, no notifications reported exposures occurring in Cherbourg, a discrete First Nations community. Taken together, these findings suggest a lower proportion of reported potential ABLV exposures among First Nations people. However, this should be interpreted cautiously given that 6.3% of cases had unknown or unstated First Nations status, as well as potential differences in behaviours related to bat–human interaction, reporting behaviour, and healthcare access. Further studies are warranted to better understand these observed differences.

No statistically significant spatio-temporal clusters were detected under the selected scan parameters. The variation and recent increase in notifications may instead reflect broader changes such as increased human–bat interactions. While there were no major changes in surveillance or notification systems during the study period, ongoing public health messaging and clinician education regarding ABLV may have contributed to greater awareness of bat-related exposure risks, potentially influencing healthcare-seeking behaviour and reporting practices over time.

The seasonal pattern of exposure incidents, with peaks in spring and summer and troughs in winter, aligned well with previous findings from metropolitan Queensland [24] and known ecological and behavioural patterns of Australian bats. Black- and grey-headed flying-foxes share similar reproductive cycles, with mating in autumn and birthing in spring, while little red flying-foxes mate in late spring and give birth in early autumn [2]. Increased bat activity during these periods thus increase opportunities for human contact. Heat stress events during the summers may further elevate exposure risk, as heat-induced debilitation and disorientation can lead to increased numbers of bats falling or descending to the ground where they are more likely to come into contact with people [31]. Conversely, the reduced notifications during winter were consistent with decreased bat activity from torpor or hibernation [32,33].

The exposure incidents were concentrated in the eastern LGAs and closely overlapped with known geographic distribution of bats. The increase in notifications in 2025 was primarily driven by incidents in Toowoomba, South Burnett and Western Downs. Notably, pre-positioned PEP is currently available at several hospital EDs in Toowoomba, South Burnett and Southern Downs, but not in Western Downs. Given the ongoing occurrence of exposures in Western Downs and its distance from facilities where PEP is available, establishing local PEP availability could facilitate more timely access and reduce logistical demands on both patients and PHU staff.

A significant increase in occupational exposures, particularly among bat rescuers, was observed in recent years, accompanied by a reduction in the proportion of exposures involving members of the general public. One possible explanation is that individuals encountering bats are increasingly seeking assistance from trained bat rescuers rather than handling the bats themselves. However, as data on bat rescue activities were not available for this study, their contribution to the observed increase in occupational exposures could not be assessed. Importantly, all occupational exposure cases notified in 2025 had previously received pre-exposure prophylaxis, meaning that post-exposure management typically involved only two vaccine doses and no HRIG. This highlights the protective value of pre-exposure vaccination among high-risk groups.

Despite ongoing public health messaging emphasising “do not touch bats” [34], intentional contact with bats by members of the general public remained the leading cause of exposure throughout the study period and accounted for more than half of notifications, although a modest reduction was observed in recent years. Previous studies have shown that while awareness of ABLV and perceived health risk associated with bat contact are generally high among Queenslanders, intentional contact is often motivated by concern for animal welfare or attempts to protect pets or family members [35,36,37,38]. These findings suggest that awareness of risk alone may be insufficient, and that future communication strategies may need to address the underlying emotional and situational drivers rather than relying solely on risk-based messaging. For example, evidence suggests that messages highlighting the distress to bats during attempted rescue, rather than human health risks, may be more effective in influencing behavioural changes [36].

The reduction in cases requiring five doses of rabies vaccine between 2011–2015 and 2016–2020 reflects revisions to prevailing national recommendations, whereby the recommended PEP schedule was reduced from five to four doses for immunocompetent individuals in 2012 [39,40]. However, the current four-dose regimen, with fixed days of administration (days 0, 3, 7 and 14), may still present logistical challenges for some patients, particularly in rural settings where travel to EDs or GPs may be required on work or school days. Greater flexibility in recommended vaccination timing could potentially alleviate some of these barriers. For example, the World Health Organization (WHO) guidelines recommend that the fourth dose may be administered between day 14 and day 28 after the first vaccine dose [41].

Of note, the apparent improvement in documented PEP completion over time should be interpreted cautiously, as the proportion of notifications with an unknown PEP outcome was substantially higher in the earlier study period compared to later periods. Thus, improved completeness of documentation may account for part of the observed increase.

### 4.2. Existing Gaps and Operational Challenges

Although the timeliness of response, reflected in the time from exposure to notification and initiation of PEP, improved over the study period, the median time required by PHU staff to manage each notification increased substantially in 2021–2025. As noted by staff, management of potential ABLV exposure is inherently time-critical and requires close coordination between PHU, clinicians, and patients to ensure timely initiation of PEP. This increase in management time likely reflects growing case complexity and operational challenges identified in this study.

One contributing factor is the increasing number of atypical exposure scenarios that required careful assessment by PHU staff. An increasing proportion of notifications involved situations where no visible wound was identified, for example, microbat bites that may be difficult to detect [42], healed wounds from delayed presentation, or pre-existing skin conditions such as dermatitis at the site of contact, which made determination of exposure challenging. Some exposure circumstances also involved indirect or uncertain contact with bats, such as a bat found inside a room where someone has been sleeping. Although such “cryptogenic” transmission of lyssavirus has been reported, the mechanism remains uncertain [43,44,45] and explicit guidance for managing such scenarios is limited in current CDNA guidelines. These atypical situations required cautious professional assessment, and staff employed a precautionary approach by managing them as potential exposures requiring PEP.

Similarly, while current Australian recommendations clearly emphasise avoiding administration of HRIG and the first vaccine dose at the same anatomical site to prevent cross-neutralisation, guidance on site selection for subsequent rabies vaccine doses was less explicit, including whether administration on the same side is appropriate and how to manage bilateral wound scenarios where HRIG has been administered locally to both sides. International guidance on this issue varies. For example, guidelines from the United Kingdom (UK) recommend alternating deltoids for subsequent doses [46], whereas the United States guidelines allow them to be administered at the same anatomical location where HRIG was previously given [47]. Clearer national guidance could help reduce uncertainty and variation in practice. While Australian and United States guidance recommend routine systemic intramuscular administration of any remaining HRIG dose after local infiltration, this is no longer recommended by the WHO [41] or the UK [48] due to limited evidence of additional clinical benefit and cost considerations [49]. Of note, these international recommendations refer to rabies broadly and are not ABLV-specific, although they also include bat-associated rabies, with ABLV management in Australia largely following these recommendations.

In addition, uncertainty regarding the use of rabies serology sometimes arose in practice. Current CDNA guidelines recommend serological testing after completion of PEP primarily for immunocompromised individuals or for patients with significant HRIG overdosing. However, in practice, serology is sometimes used to assess immune response when deviations from recommended vaccination schedules occur, which is not uncommon [50,51]. While demonstration of adequate rabies antibody level may provide reassurance to both clinicians and patients, the exact serological correlate of protection against rabies in humans, particularly for ABLV, is not well established [52]. A recent study also showed that failed seroconversion might occur following deviation from recommended PEP regimens and among older age groups [53]. Thus, clearer guidance on the circumstances under which serology may be appropriate could therefore support more consistent and efficient case management.

Lastly, the proportion of notifications in which the involved bat was tested for ABLV declined significantly over the study period. Current CDNA guidelines recommend testing the implicated bat when feasible. However, as described by PHU staff, bat testing in practice was often limited by the availability of the bat and logistical constraints including cost, transport requirements, and biosafety considerations. In many cases, the bat was not retrieved (e.g., had flown away or was already disposed of) or was not in a good diagnostic condition (e.g., not refrigerated soon after death) for testing at the time of notification [54]. While bat testing can reduce unnecessary vaccine doses and provide reassurance when results are negative, or reinforce adherence when positive [55], the operational burden may limit its feasibility in many circumstances. Clearer national or state guidance on the operational aspects of bat testing, including responsibilities for specimen handling, transport logistics, and funding arrangements, may help improve the feasibility and consistency of testing practices.

### 4.3. Implications for Service Planning and Improvement

Based on the findings of this study, several measures could strengthen preparedness and operational efficiency for managing potential ABLV exposure. Expanding availability of pre-positioned PEP to Western Downs would improve timely access and reduce logistical burdens for both patients and staff. Operationally, this would first involve engaging the local public hospital with exposure data and service needs from the region to secure organisational buy-in. Subsequent implementation would involve establishing arrangements for PEP delivery and storage, developing local clinical pathways for case management, training staff in case assessment and PEP administration, and establishing standardised documentation and communication processes between the hospital and DDPHU. Continuing to promote pre-exposure vaccination, safe bat handling practices, and appropriate use of personal protective equipment among high-risk groups, such as bat rescuers, remains essential given the increasing number of exposures in this population. Public health messaging should be refined to address the underlying motivations behind intentional bat contact and complement traditional risk-based communications. While awaiting updates to CDNA guidelines, DDPHU should develop internal, evidence-based protocols to guide case management regarding vaccine administration sites, serology use, local bat testing processes, and atypical scenarios such as cryptogenic exposure. Finally, ongoing monitoring of potential ABLV exposure notifications and staff workload will be important to ensure workforce sustainability. Future studies could evaluate the feasibility, effectiveness, and impact of these measures on exposure prevention and service delivery.

Of note, the operational challenges and proposed measures identified in this study are also relevant to other public health units in Australia and other countries managing potential bat-associated lyssavirus exposures, particularly those serving rural and geographically dispersed populations. More broadly, these findings may inform rabies and other lyssavirus prevention programs globally, particularly in rural and remote contexts. For example, geographic and logistical barriers to timely PEP access may be reduced through strategic pre-positioning of rabies vaccines and HRIG at geographically accessible health facilities. This should be supported by clear local clinical pathways, standardised documentation and communication processes, and coordination between public health services and healthcare providers. Nevertheless, the applicability of these approaches will depend on the local epidemiology and health-system capacity. In the Australian setting, barriers related to timely access to PEP may delay but do not typically prevent eventual administration. In contrast, in many low-resource settings, these same barriers can result in incomplete or no access to PEP following exposure [56]. This has important implications for ensuring timely and equitable delivery of care and underscores the critical role of health system capacity in preventing rabies and lyssavirus-related mortality.

### 4.4. Strengths and Limitations

This study adopts a mixed-methods approach combining long-term surveillance data with qualitative insights from PHU staff, providing both epidemiological and operational perspectives on managing potential ABLV exposures in regional, rural and remote Queensland. Analysis of temporal and spatial patterns further enhances understanding of exposure trends and informs service planning.

However, several limitations should be acknowledged. Not all potential exposures might have been notified to DDPHU, and some exposures occurring within the region might have been managed by other PHUs if the case resided outside the DDPHU catchment. The qualitative findings were based on records of three CQI meetings, which might not have captured the full range of experiences or challenges encountered. These findings reflect the experiences of DDPHU staff and may not be transferable to other PHUs with different workflows or contexts. Similarly, the overall study findings may not be generalisable to other jurisdictions due to differences in geography, healthcare access, and human–bat interaction patterns. Additionally, the time spent managing cases was self-reported by staff and may be subject to recording error or incomplete documentation, particularly for cases in the earlier study period. The analysis also did not account for contextual factors that may influence notification management and exposure-related behaviours, such as staff availability, time or day of notification, PEP availability, competing PHU priorities, and concurrent health messaging and alerts during high-risk seasons. Data on factors associated with delays in notification or administration of PEP were not collected and therefore could not be assessed. The relatively small number of cases may also have limited the statistical power of the spatio-temporal cluster analysis. The bat distribution data captures only the spatial extent of bat presence and does not include count estimates, thus precluding quantitative correlation with exposure notifications. Finally, given the retrospective study design and reliance on routinely collected data, findings may be affected by missing data (particularly in the earlier years), potential underreporting of exposures, and changes in management guidelines and PEP schedules over the study period, which may have influenced case notification and epidemiological trends.

## 5. Conclusions

This mixed-methods study provides a comprehensive overview of potential ABLV exposure in the Darling Downs and South West regions of Queensland from 2011 to 2025. Overall, although the epidemiology of exposures aligned with known bat distribution and seasonal activity, the increase in notifications in 2025 has contributed to greater workload demands. Staff from DDPHU identified several operational challenges arising from the time-critical nature of case management, including increasing case complexity, atypical exposure scenarios and aspects of practice not explicitly addressed in current national guidelines. Integrating these findings enabled the development of several actionable recommendations to strengthen preparedness, enhance operational efficiency, and support workforce sustainability for managing potential ABLV exposure within DDPHU. These findings may also inform public health responses to lyssavirus exposure in other non-urban settings with barriers to timely PEP access.

## Figures and Tables

**Table 1:** Study objectives, data sources, and metrics.

Objectives	Data Sources	Metrics
Describe the epidemiology of potential ABLV exposure notifications to DDPHU between 2011 and 2025	Notifiable Conditions System (NoCS) DDPHU case follow-up records BatMap database	Descriptive epidemiology of notifications Temporal and spatial trends in notifications
2. Identify operational challenges encountered by DDPHU staff in managing potential ABLV exposure notifications	DDPHU meeting records	Key operational challenges
3. Inform service planning and targeted public health interventions in managing potential ABLV exposure notifications	Synthesised findings from quantitative and qualitative analysis	Identification of service gaps Recommendations for service improvement

**Table 2:** Demographics, exposure characteristics, and management of potential ABLV exposure notifications, 2011–2025.

Characteristic *	2011–2015 (*n* = 75)	2016–2020 (*n* = 75)	2021–2025 (*n* = 87)	Overall (*n* = 237)	*p*-Value
**Age (year)**	47 (26, 58)	48 (32, 60)	45 (29, 61)	46 (29, 59)	0.47 ^†^
**Sex**					
Male	30 (40%)	44 (59%)	45 (52%)	119 (50%)	0.07 ^§^
Female	45 (60%)	31 (41%)	42 (48%)	118 (50%)	
**Indigenous status**					
First Nations	1 (1.3%)	3 (4.0%)	3 (3.4%)	7 (3.0%)	0.70 ^¶^
Non-First Nations	70 (93%)	71 (95%)	74 (85%)	215 (91%)	
Unknown/Not stated	4 (5.3%)	1 (1.3%)	10 (11%)	15 (6.3%)	
**Time of day of exposure**					
Morning (06:00–11:59)	23 (31%)	16 (21%)	25 (29%)	64 (27%)	0.49 ^¶^
Afternoon (12:00–17:59)	18 (24%)	17 (23%)	16 (18%)	51 (22%)	
Evening (18:00–23:59)	20 (27%)	29 (39%)	22 (25%)	71 (30%)	
Overnight (00:00–05:59)	4 (5.3%)	8 (11%)	8 (9.2%)	20 (8.4%)	
Unknown	10 (13%)	5 (6.7%)	16 (18%)	31 (13%)	
**Local Government Area of exposure**					
Toowoomba	21 (28%)	34 (45%)	42 (48%)	97 (41%)	<0.05 ^¶^
Southern Downs	17 (23%)	7 (9.3%)	12 (14%)	36 (15%)	
South Burnett	6 (8.0%)	11 (15%)	13 (15%)	30 (13%)	
Western Downs	10 (13%)	9 (12%)	11 (13%)	30 (13%)	
Goondiwindi	3 (4.0%)	4 (5.3%)	0 (0%)	7 (3.0%)	
Cherbourg	0 (0%)	0 (0%)	0 (0%)	0 (0%)	
Maranoa	3 (4.0%)	2 (2.7%)	1 (1.1%)	6 (2.5%)	
Balonne	3 (4.0%)	1 (1.3%)	0 (0%)	4 (1.7%)	
Murweh	2 (2.7%)	0 (0%)	0 (0%)	2 (0.8%)	
Paroo	0 (0%)	0 (0%)	1 (1.1%)	1 (0.4%)	
Bulloo	0 (0%)	0 (0%)	0 (0%)	0 (0%)	
Quilpie	0 (0%)	0 (0%)	0 (0%)	0 (0%)	
Unknown	10 (13%)	7 (9.3%)	7 (8.0%)	24 (10%)	
**Animal involved in exposure**					
Flying Fox	37 (49%)	52 (69%)	57 (66%)	146 (62%)	<0.01 ^¶^
Microbat/Insectivorous Bat	31 (41%)	21 (28%)	18 (21%)	70 (30%)	
Bat (unspecified)	1 (1.3%)	2 (2.7%)	10 (11%)	13 (5.5%)	
Other	4 (5.3%)	0 (0%)	0 (0%)	4 (1.7%)	
Unknown	2 (2.7%)	0 (0%)	2 (2.3%)	4 (1.7%)	
**Circumstances of exposure**					
General public, accidental contact with bats	23 (31%)	17 (23%)	12 (14%)	52 (22%)	<0.01 ^¶^
General public, intentional contact with bats ^‡^	38 (51%)	55 (73%)	50 (57%)	143 (60%)	
General public, no direct contact with bats	2 (2.7%)	0 (0%)	1 (1.1%)	3 (1.3%)	
Occupational contact with bats	6 (8.0%)	3 (4.0%)	24 (28%)	33 (14%)	
Unknown/Others	6 (8.0%)	0 (0%)	0 (0%)	6 (2.5%)	
**Type of exposure**					
Bite and/or scratch	65 (87%)	73 (97%)	76 (87%)	214 (90%)	<0.01 ^¶^
Contact with no visible wound identified or with pre-existing wound	2 (2.7%)	1 (1.3%)	7 (8.0%)	10 (4.2%)	
Other or unknown exposure	8 (10.7%)	1 (1.3%)	4 (4.6%)	13 (5.5%)	
**Animal ABLV test result**					
Not tested	56 (75%)	70 (93%)	81 (93%)	207 (87%)	<0.01 ^¶^
Tested-Negative/Equivocal	10 (13%)	4 (5.3%)	1 (1.1%)	15 (6.3%)	
Tested-Positive	7 (9.3%)	0 (0%)	1 (1.1%)	8 (3.4%)	
Unknown	2 (2.7%)	1 (1.3%)	4 (4.6%)	7 (3.0%)	
**Required PEP**	66 (88%)	72 (96%)	87 (100%)	225 (95%)	<0.01 ^¶^
**Number of rabies vaccine doses required**					
Not required	9 (12%)	3 (4.0%)	0 (0%)	12 (5.1%)	<0.01 ^¶^
2	6 (8.0%)	7 (9.3%)	26 (30%)	39 (17%)	
4	40 (53%)	65 (87%)	60 (69%)	165 (70%)	
5	19 (25%)	0 (0%)	1 (1.1%)	20 (8.5%)	
Unknown	1 (1.3%)	0 (0%)	0 (0%)	0 (0%)	
**Received ≥1 rabies vaccine dose**	49 (65%)	72 (96%)	85 (98%)	206 (87%)	<0.01 ^¶^
**Required HRIG**					
Yes	53 (71%)	63 (84%)	55 (63%)	171 (72%)	<0.05 ^§^
Unknown	3 (4.0%)	0 (0%)	0 (0%)	3 (1.3%)	
No	19 (25%)	12 (16%)	32 (37%)	63 (27%)	
**Received required HRIG**					
Yes	17 (23%)	62 (83%)	53 (61%)	132 (56%)	<0.01 ^¶^
Not required	19 (25%)	12 (16%)	32 (37%)	63 (27%)	
Unknown	39 (52%)	1 (1.3%)	2 (2.3%)	42 (18%)	
**Days from exposure to notification**	5 (0, 678)	1 (0, 13)	1 (0, 31)	1 (0, 678)	<0.01 ^†^
**Days from exposure to first rabies vaccine dose**	2.0 (0.0, 658.0)	1.0 (0.0, 14.0)	1.0 (0.0, 35.0)	1.0 (0.0, 658.0)	<0.01 ^†^
**Days from exposure to HRIG administration**	1.00 (0.00, 29.00)	1.00 (0.00, 7.00)	1.00 (0.00, 38.00)	1.00 (0.00, 38.00)	0.13 ^†^
**PEP outcome**					
Completed	22 (29%)	67 (89%)	83 (95%)	172 (73%)	<0.01 ^¶^
Discontinued because bat tested negative	1 (1.3%)	1 (1.3%)	1 (1.1%)	3 (1.3%)	
Not required because bat tested negative	9 (12%)	3 (4.0%)	0 (0%)	12 (5.1%)	
Refused by case	0 (0%)	0 (0%)	2 (2.3%)	2 (0.8%)	
Unknown	43 (57%)	4 (5.3%)	1 (1.1%)	48 (20%)	
**Case management duration by PHU staff (minutes)**	110 (80, 153)	85 (70, 115)	160 (100, 220)	110 (78, 170)	<0.01 ^†^

* Data are presented as medians (with IQR in parentheses) for continuous variables and n (%) for categorical variables. Percentages are calculated using the total number of cases in the corresponding 5-year period as the denominator; ^†^ Kruskal–Wallis rank sum test; ^§^ Pearson’s Chi-squared test; ^¶^ Fisher’s Exact Test; All statistical tests assess whether the distribution of cases across categories differs by the three time periods. ^‡^ This category also included a small number of cases involving intentional removal of bats from domestic animals (e.g., cats or dogs).

**Table 3:** Summary of themes regarding operational challenges in managing potential ABLV exposures.

SEIPS Domains	Themes	Descriptions
Person	Workload and urgency	Managing a potential ABLV exposure case can involve substantial workload, as each case is highly variable and not always straightforward; Potential ABLV exposure notifications are time-critical and are prioritised to ensure timely assessment and access to PEP.
	Professional judgement and guidance	Existing guidelines address most common exposure scenarios; however, some special situations are not explicitly covered, for example: ○ Cryptogenic exposures (e.g., bat found in a room where someone has been sleeping, without known contact); ○ Contact with no visible wound; ○ Contact over a pre-existing wound; ○ Which deltoid for subsequent rabies vaccine administration following HRIG administration; ○ Use of rabies serology in vaccine administration deviations. These situations require staff to apply professional judgement to decision-making. Staff often err on the side of caution.
Processes and tasks	Case complexity and coordination	Case management involves multiple steps, including risk assessment, client follow-up, coordination with clinicians, and monitoring PEP completion; The complexity and variability of cases can significantly increase administrative and coordination demands.
	Follow-up and continuity of care	Clients may move between locations or change providers during the PEP course, thus requiring additional effort and follow-up to ensure vaccine availability and completion of vaccination schedules; Communications with clients, hospital emergency departments, and general practice clinics account for a significant portion of the workload; Strict scheduling requirements for rabies vaccine doses under the current guidelines can create logistical challenges, particularly when doses fall on public holidays or weekends with limited general practitioner (GP) availability. Patients in rural or outer regional areas may also face long travel times and extended waiting periods in emergency departments.
Technology and tools	Record management and data systems	Rabies vaccine administered in public hospitals currently requires manual entry into AIR, unlike in GP settings where the process is automated through practice management software. This requires extensive follow-up by PHU staff to ensure accurate documentation on AIR.
Organisation	Access to pre-positioned PEPs	Pre-positioned rabies vaccines and HRIG at selected hospitals support timely access to PEP; Nevertheless, for patients residing outside those areas, accessibility is still an issue, as they sometimes need to travel a few hours to receive treatment.
	Resource allocation and service capacity	Bat testing is mentioned in the current guidelines as it can inform decision-making on whether PEP is/is still required. However, organising bat testing is resource-intensive, e.g., logistics to have the bat transported for testing, funding for transport and testing fees.

## Data Availability

Data are available on request due to privacy and ethical restrictions.

## Abbreviations

The following abbreviations are used in this manuscript:

ABLV	Australian bat lyssavirus
PEP	Post-exposure prophylaxis
DDPHU	Darling Downs Public Health Unit
HRIG	Human rabies immunoglobulin
PHU	Public health unit
LGAs	Local government areas
CDNA	Communicable Diseases Network Australia
ED	Emergency department
GP	General practitioner
AIR	Australian Immunisation Register
NoCS	Notifiable Conditions System
CQI	Continuous Quality Improvement
SEIPS	Systems Engineering Initiative for Patient Safety
WHO	World Health Organization
UK	United Kingdom
